# Application Research of Individualized Conditional Reprogramming System to Guide Treatment of Gastric Cancer

**DOI:** 10.3389/fonc.2021.709511

**Published:** 2021-07-16

**Authors:** Weizhu Zhao, Kai Liu, Zhikun Sun, Longgang Wang, Bing Liu, Luguang Liu, Xianlin Qu, Zhixiang Cao, Jujie Sun, Jie Chai

**Affiliations:** ^1^ Department of Radiology, Cancer Hospital Affiliated to Shandong First Medical University, Shandong Cancer Hospital and Institute, Jinan, China; ^2^ Department of Oncology, Binzhou People’s Hospital, Binzhou, China; ^3^ Department of Gastrointestinal Surgery, Cancer Hospital Affiliated to Shandong First Medical University, Shandong Cancer Hospital and Institute, Jinan, China; ^4^ Department of Urinary Surgery, Zhaoyuan People’s Hospital, Zhaoyuan, China; ^5^ Department of Postgraduate, Shandong First Medical University, Jinan, China; ^6^ Department of Research and Development, Beijing Percans Oncology Co. Ltd., Beijing, China; ^7^ Department of Pathology, Cancer Hospital Affiliated to Shandong First Medical University, Shandong Cancer Hospital and Institute, Jinan, China

**Keywords:** gastric cancer, individualized conditional reprogramming, chemotherapy, drug sensitivity, individual treatment

## Abstract

**Background:**

Gastric cancer (GC) is one of the most common causes of malignant tumors in the world. Due to the high heterogeneity of GC and lack of specificity of available chemotherapy regimens, these tumors are prone to resistance, recurrence, and metastasis. Here, we formulated an individualized chemotherapy regimen for GC using a modified individual conditional reprogramming (i-CR) system. We established a primary tumor cell bank of GC cells and completed drug screening in order to realize individualized and accurate GC treatment.

**Methods:**

We collected specimens from 93 surgical or gastroscopy GC cases and established a primary tumor cell bank using the i-CR system and PDX models. We also completed *in vitro* culture and drug sensitivity screening of the GC cells using the i-CR system. Whole-exome sequencing (WES) of the i-CR cells was performed using P0 and P5. We then chose targeted chemotherapy drugs based on the i-CR system results.

**Results:**

Of the 72 cases that were collected from surgical specimens, 26 cases were successfully cultured with i-CR system, and of the 21 cases collected from gastroscopy specimens, seven were successfully cultured. Among these, 20 cases of the PDX model were established. SRC ± G3 had the highest culture success rate. The i-CR cells of P0 and P5 appeared to be highly conserved. According to drug sensitivity screening, we examined the predictive value of responses of GC patients to chemotherapeutic agents, especially in neoadjuvant patients.

**Conclusion:**

The i-CR system does not only represent the growth characteristics of tumors *in vivo*, but also provides support for clinical drug use. Drug susceptibility results were relatively consistent with clinical efficacy.

## Introduction

Gastric cancer(GC)is one of the most common causes of malignant tumors in the world. There were about 100,000 new cases of GC, and 780,000 deaths worldwide in 2018, which ranks it third in malignant tumors (1). The incidence of GC is significantly higher in East Asia and South America than in other regions of the world. However, more than 80% of advanced GC cases are found in China, with large gaps between rates there and rates in South Korea and Japan (2, 3). Additionally, the five-year survival rate for GC is relatively low. At present, GC treatment still depends on surgery, chemotherapy, and radiotherapy. Targeted therapy and immunotherapy have brought benefits to some patients, but the results are still not promising for most patients. There are many chemotherapy options for GC because of its high degree of heterogeneity, but the lack of specificity of available treatments can lead to resistance, recurrence, and metastasis (4). Thus, formulating individualized GC chemotherapy regimens is an urgent problem for clinical treatment of GC.

At present, the most widely used methods for clinical drug sensitivity detection are gene sequencing and immunohistochemistry. However, these methods are also limited by indirectness and uncertainty. Chemosensitivity in cell culture alone often cannot recreate microenvironments or tumor heterogeneity in tumor tissues, so the results are often not accurate. At present, the model of “human tumor tissue xenotransplantation (PDX)” is the most recognized in the world (5–7). In this model, a small piece of tumor tissue taken from the patient is implanted into an experimental mouse to simulate its original growth environment, thus preserving the characteristics of the patient and the tumor to the maximum extent. However, the PDX model has some disadvantages, including low tumor formation rate, long methodological cycle, and high costs (8, 9). Additionally, its benefits to patients have not been demonstrated in a clinical environment.

Conditional reprogramming (CR) is a new *in vitro* culture system that combines a feeding cell system and a Rho-associated kinase (ROCK) inhibitor (10–12). The limitation of CR technology is that it cannot distinguish between tumor cells and normal epithelial cells, because both proliferate well in the system. Recently, however, an improved individual CR (i-CR) system has been developed, which is characterized by the selective expansion of tumor cells cultured *in vitro* from patients with colorectal cancer (CRC) (13). The i-CR system can screen out effective individualized drugs in a short time using the innovative technology of high connotation analysis and an associated detection system, which is rapid, efficient and has the capacity for high-throughput drug sensitivity detection *in vitro*. This system can nominate individualized chemotherapy regimens, which may both improve treatment effectiveness and lower costs (14–16). Thus, the i-CR system has good prospects in personalized cancer treatment and translational medicine (17), but it has not been applied in GC.

Here, we successfully established a primary tumor cell bank of GC cells, completed drug screening using the i-CR system, and guided neoadjuvant and postoperative adjuvant therapy of GC patients, realizing individualized and accurate treatment for GC.

## Patients and Methods

**Figure 1:**
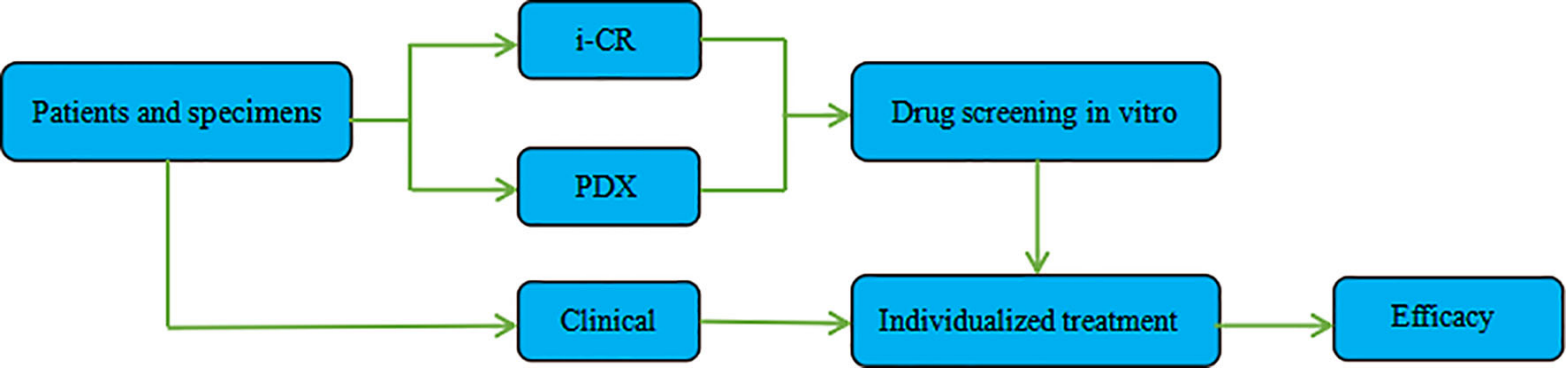
Specific method and flow chart of the i-CR system and PDX platform.

Combining the i-CR system and PDX platform and using GC specimens obtained *via* surgical or gastroscopy methods, we established a tumor chemotherapeutic drug sensitivity evaluation system, formulated an individualized chemotherapy regimen, and conducted a systematic evaluation of its efficacy. The specific process and methods are shown in (**Figure 1** **)**.

### Collection of GC Specimens

We collected surgical or gastroscopy specimens according to requirements for material collection, washed the specimens with sterile physiological saline at 4°C several times to prevent contamination, quickly placed the specimens into a 4°C preservation liquid tube and molded the chain to the technology platform.

### Pretreatment of the Establishment of GC Primary Tumor Cell Bank

The received GC specimens were washed twice with PBS at 4°C and sectioned in a sterile Petri dish using surgical scissors. Specimens were then subjected to enzymatic dissociation with a combination of collagenase I, DNase and dispase. Final cell suspensions were filtered through 100 μm cell strainers, followed by pelleting and resuspension in the complete i-CR medium.

### Establishment of GC Primary Tumor Cell Bank

1. Preparation of feeder cells: NIH3T3 fibroblasts were treated with mitomycin C (MMC) at concentrations of 1–20 μg/ml for 2 h at 37°C. The cells were then digested, and the cell pellets were frozen for further testing.
2. Growth curve determination and plating of feeder cells: The mitomycin C-treated cells were checked for their stalled proliferation with standard MTT method. NIH3T3 cells lethally irradiated at 40 Gy were used as a comparison. The results were shown in **Figure S1**. As results from mitomycin C treatment at concentrations above 5 μg/ml were comparable to irradiation, 10 μg/ml was chosen for routine use. After resuscitation, feeder cells that passed the cryopreservation test were plated in a cell culture plate at a certain density. They were used after being attached to the wall for 24 h.
3. Collection of sample cells: Cultured sample cells from the culture flask were digested with trypsin, and serum-containing medium was added for neutralization. Cells were collected in a 50ml centrifuge tube and centrifuged at 1,000 rpm for 5 min. After centrifugation, the supernatant was removed, leaving the precipitate.
4. Resuspension of the sample cells: The collected cell pellet was resuspended in the culture medium, pipetted evenly, and centrifuged at 1,000rpm for 5min. After centrifugation, the supernatant was removed, leaving the pellet, which was then resuspended in a plating medium.
5. Counting of sample cells: A certain amount of cell suspension was taken and diluted to a total volume of 100 μl (100 μl cell fluid + 0.2 μl G + 1 μl H).
6. Sample cell plating: According to the obtained counting results, the required number of cells was calculated and added to the plating medium. This combination was mixed well and spread added to feeder cells in the corresponding wells. The next phase of the drug screening experiment proceeded following next-day observation.

### Establishment of PDX Models

The received GC specimens were subcutaneously inoculated into immunodeficient mice. After the tumor grew to 1,000mm^3^ in the mouse, the tumor tissue (P0) was surgically removed, and then cut into small tumor pieces with a diameter of 3 mm × 3 mm under sterile conditions. Each small piece of tumor tissue was transplanted into a new immunodeficient mouse for *in vivo* passage. All 1,000 mm^3^ tumor tissue pieces were passed through 5–10 mice. These passaged tumor tissues (P1) continued to be passaged after growing to 1,000mm^3^ to ensure the integrity of the model. When the passage of tumor cells reached P1, a part of the tumor mass was permanently frozen with liquid nitrogen as a model seed bank for subsequent project research.

### Drug Screening With the i-CR System

**Figure 2:**
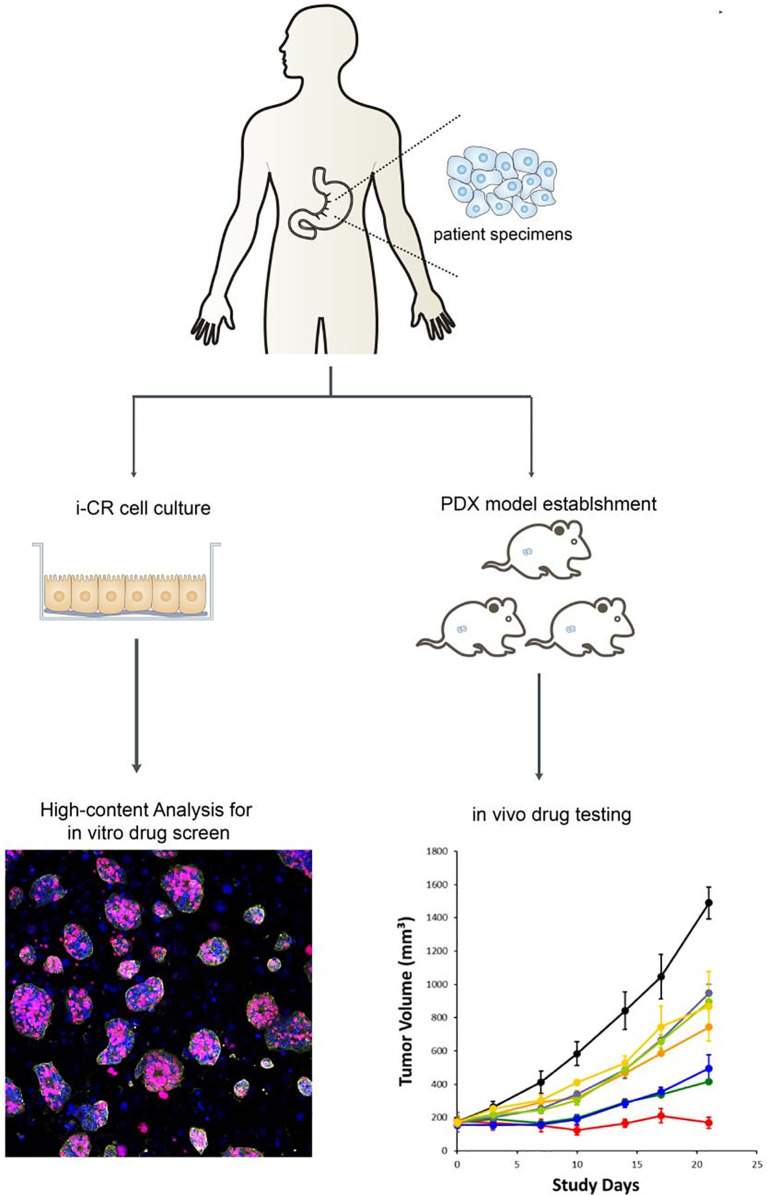
*In vitro* culture and drug sensitivity screening process of GC cells using the i-CR system and PDX model.

After tumor cells were plated for 24 h, treatments—using different concentrations and doses of drugs—began. The original media were aspirated in the wells, and 200 ul of fresh media containing drugs were added. After that, the cells were returned to the incubator and continued to be cultured for seven days. Then, after drug elution, proliferation labeling, staining, and high content analysis, the total number of tumor cells and the number of proliferating tumor cells at each drug concentration were analyzed to obtain the percentage of tumor cell proliferation in each condition (**Figure 2**).

**Figure 3:**
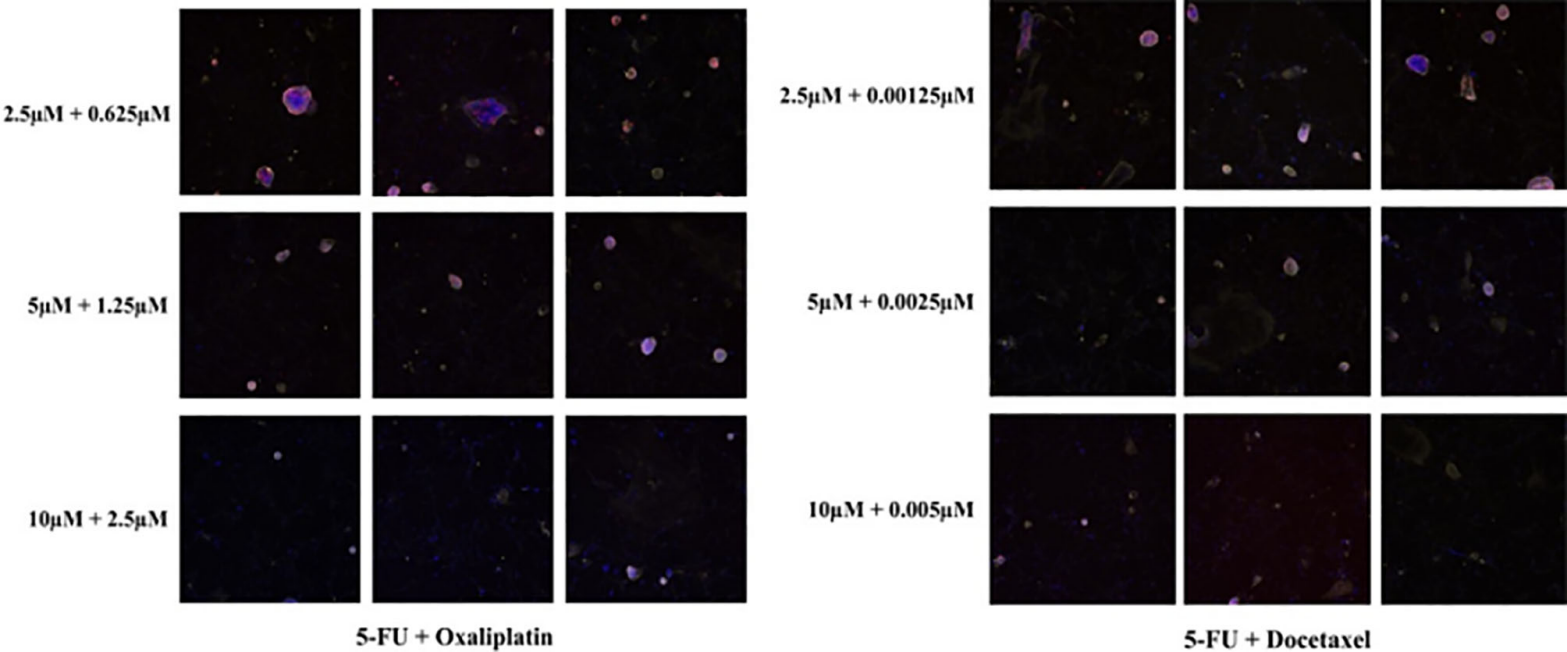
*In vivo* drug sensitivity tests of different drug concentrations in the i-CR system. MI and DSI values were calculated using formulas. (The specimens were from NYL-JN-129).

The effectiveness of each therapeutic regimen was evaluated and quantified using the following formulas: 1) Maximum Inhibition (MI) = N_0_/N_d_, where N_0_ and N_d_ denote the number of EpCAM + EdU + epithelial cells in control wells or in the wells with drug concentrations at C_0_, respectively. A larger MI value represents stronger inhibitory effects of the drug on tumor cell growth at the area under the drug-time curve (AUC) concentrations. 2) Drug Sensitivity Index (DSI) = 1/4Ln(MI_C0_) + 1/2Ln(MI _1/2C0_) + Ln(MI_1/4C0_), where MI_C0_, MI_1/2C0_, and MI_1/4C0_ are the MI values observed when cells were treated at drug concentrations C_0_,1/2C_0_ and 1/4C_0_, respectively (**Figure 3**). The larger the DSI value, the better the inhibitory effect of the drug compared with other drugs (14).

### Whole-Exome Sequencing With the i-CR Cells

P0 and P5 GC cells from i-CR system were analyzed using whole-exome enrichment sequencing (WES). The outcomes were single nucleotide variations (SNVs), copy number, and mutation frequency. WES was performed as described previously (13). Control-FREEC was used to detect somatic copy-number variations (CNVs). It divided the genome into small contiguous regions using sliding windows. The read count profiles in each region for normal and tumor samples were computed and normalized accounting for GC-content and mappability. The read count ratios of tumors to matched normal samples were calculated and used as the proxy of the copy number ratios.

### Clinical Validation of Chemosensitivity Assays

This research was approved by the Shandong Cancer Hospital, which is affiliated with Shandong First Medical University. All specimens were collected from patients who gave written informed consent.

The clinical data of 93 patients with advanced esophageal–gastric junction adenocarcinoma or gastric cancer from October 26, 2018 to December 11, 2020 were collected. All patients had undergone MDT consultation, which indicated either direct surgery or neoadjuvant chemotherapy. Choice of chemotherapy drugs was based on the i-CR system results.

For surgical patients, the serum levels of CEA, CA19-9, CA74-2, and AFP were collected, and computed tomography (CT) scans of the chest, abdomen, and pelvis were performed at baseline. For neoadjuvant chemotherapy patients, the above tests were performed at baseline and were then repeated at least once every six weeks throughout the treatment regimen. Imaging examination results were evaluated according to RECIST 1.1 standards (18). Surgical specimens were accurately evaluated by experienced pathologists using American Joint Committee on Cancer (AJCC) staging standards.

Inclusion criteria for patients included: 1) clinical staging confirmed by CT, gastroscopy or ultrasound gastroscopy; 2) a KPS score >80 points; an ECOG score between 0 and 1 point; 3) measurable lesions according to RECIST 1.1 standards; 4) before-treatment neutrophil count ≥1.5 × 10^9^/L, platelet count ≥100 × 10^9^/L, hemoglobin ≥80 g/L, liver function <1.5 times the upper limit of normal, serum bilirubin ≤1.0 μmol/L, serum creatinine <1.5 μmol/L, and PT-INR/PTT <1.7 times the upper limit of normal.

Exclusion criteria included: 1) co-occurrence of serious liver, kidney, cardiovascular, or other important organ system diseases that could affect chemotherapy or surgery; 2) allergies to chemotherapy drugs and/or adjuvants; 3) receipt of any form of chemotherapy or other drugs; 4) women of childbearing age who did not agree to use contraception, as well as pregnant or lactating women; 5) patients with dysphagia, active peptic ulcers, complete or incomplete intestinal obstruction, active gastrointestinal bleeding, or perforations; 6) patients who had difficulty taking Tiggio orally; 7) patients with other types of tumors.

### Statistical Analysis

Statistical analyses were performed using SPSS 22.0 and Graphpad Prism version 6.0. Between-group differences were evaluated using the Chi-square tests, unpaired two-tailed t-test, or one-way analyses of variance (ANOVAs). A two-sided *P <*0.05 was considered statistically significant.

## Results

### Cultivation of GC Cells With the i-CR System and PDX Model

Ninety-three cases of GC were collected from October 26, 2018 to December 11, 2020, of which 72 cases were collected from surgical specimens (with twenty-six being successfully cultured), and 21 cases were collected from gastroscopy specimens (with seven being successfully cultured). Among these, 20 cases of the PDX model were established with surgical specimens, but no PDX model cases were established using gastroscopy specimens. There was no statistically significant difference in the establishment of the primary tumor cell bank between surgical specimens and gastroscopy specimens (χ^2^ = 0.055, *P* = 0.815; **Table 1**).

**Table 1:** Cultivation of GC cells with the i-CR system and PDX model.

Material type	Total number	Success of cell bank (%)	Success of PDX (%)
Surgical specimens	72	26 (36.11)	20 (27.78)
Gastroscopy specimens	21	7 (33.33)	0

**Figure 4:**
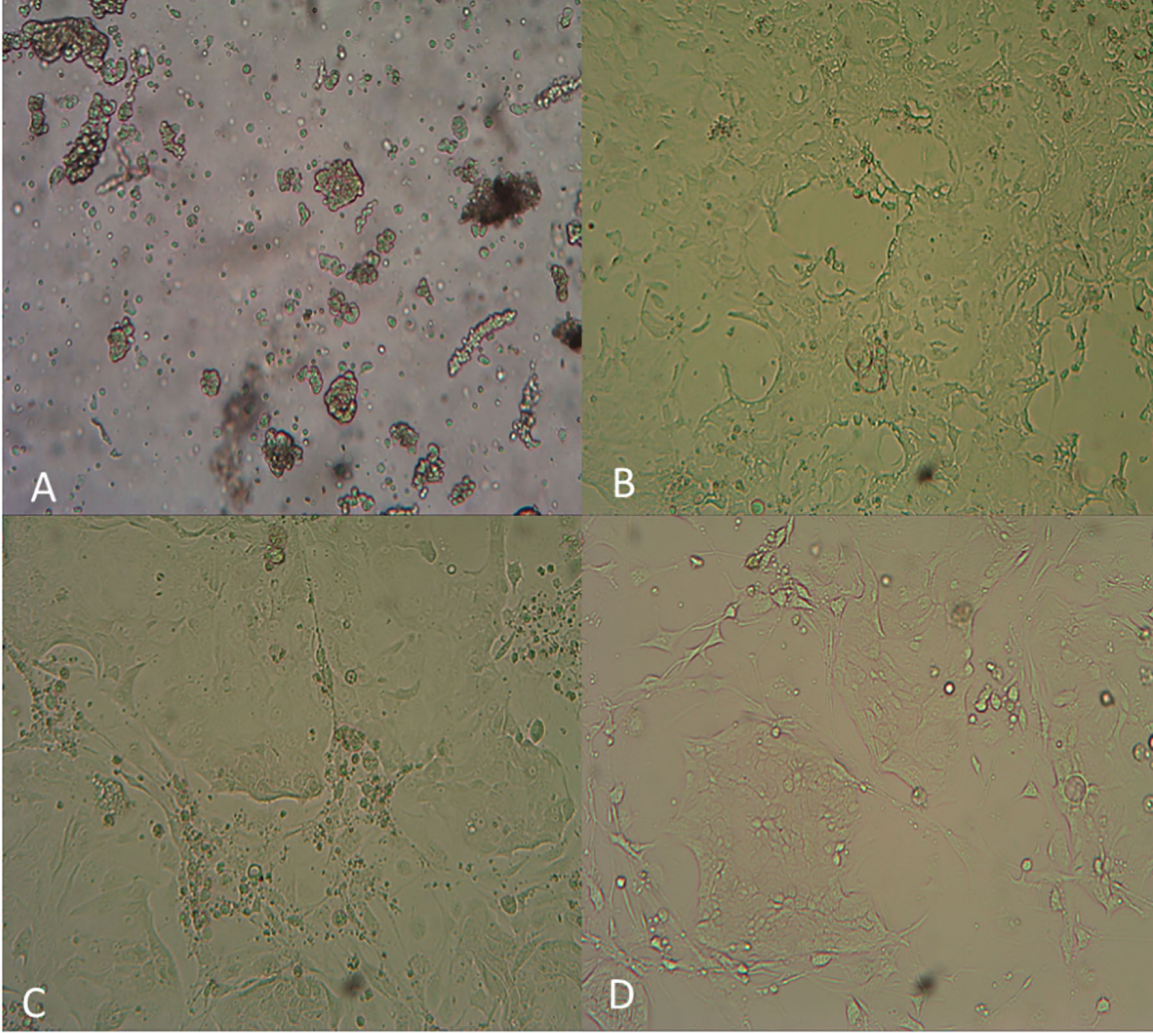
Photos of the GC primary tumor cell bank generated using the i-CR system. **(A)** Cell digestion for 30 minutes; **(B)** Cell culture for 5 days; **(C)** Cell culture for 9 days; **(D)** Cell culture for 15 days.

The GC primary cells were isolated and plated as shown in **Figure 4**. The viability of the isolated cells is monitored by Casein AM staining (**Figure S2A**). The GC tumor cells were counted as the EpCAM-positive epithelial cells (**Figure S2B**). As shown in **Figures S2C, D**, during the drug sensitivity tests, total cell numbers were marked by Hoechst staining, and living cells were displayed with EdU staining. In both cases, only the EpCAM-positive cells were figured in the final data analysis.

### Genetic Analysis of i-CR Cells

**Figure 5:**
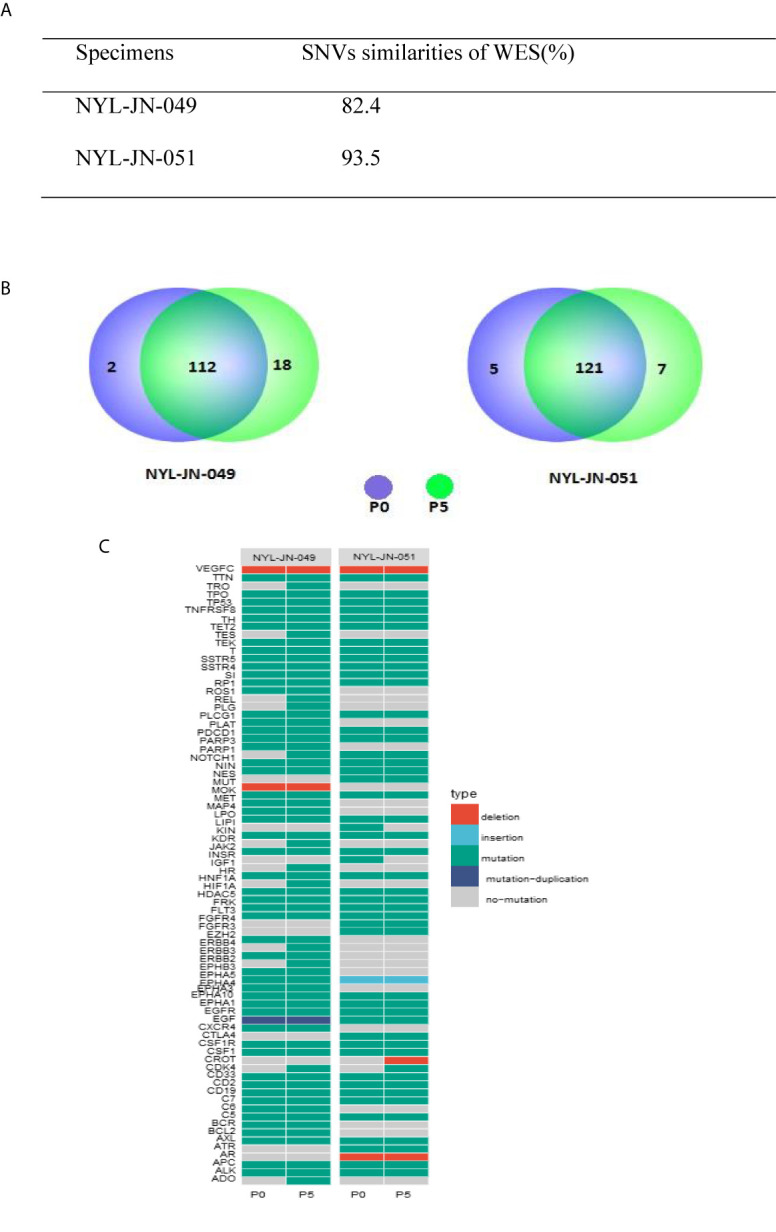
Genetic analysis of the i-CR cells. **(A)** SNV similarities between P0 and P5 i-CR cells. **(B)** Venn diagrams of SNVs in cancer-related genes for P0 and P5 i-CR cells. **(C)** Heatmap of genetic profiles of cancer-related GC genes between P0 and P5 i-CR cells.

To investigate whether i-CR cells maintained genetic heterogeneity, two pairs of specimens were tested using WES. We examined the SNVs of each specimen against the reference genome (**Figure 5**). The i-CR P0 and P5 cells from two pairs of samples (NYL-JN-049 and NYL-JN-051) shared 82.4 and 93.5% of their SNVs, respectively (**Figure 5A**). The high concordance of SNVs indicated the genomic heterogeneity was mostly maintained in the i-CR cells. This observation was also supported by comparing the SNVs of tumor-related genes (**Figure 5B**). We further analyzed genes related to GC and their expression profiles (**Figure 5C**). Next, we analyzed the copy number variations (CNVs) of samples P0 and P5. Copy number profiles of P0 and P5 were compared and summarized in **Table S1**. The results showed that they were highly conserved (<1% difference), indicating that GC i-CR cells largely maintained the genomic heterogeneity of the primary tumors. Taken together, P0 and P5 i-CR cells appeared to be highly conserved and largely maintained the genomic heterogeneity of the primary tumor cells.

### MI and DSI Guide Clinical Medication

The MI and DSI values of the therapeutic regimens for each patient are shown in (**Table 2** **)**. MI is a more intuitive indication of the inhibitory effect of each drug treatments. Higher MI values represent more effective inhibition. DSI is a novel *in vitro* drug sensitivity criteria used in this research. The calculation of DSI incorporates the populational difference of tumor cells in terms of drug sensitivity. We calculated the DSI values of the drugs using the derived mathematical formula. We then selected corresponding chemotherapy regimens based on DSI values.

**Table 2:** MI and DSI values of the therapeutic regimens for each patient.

Patients	MI			DSI		
	5-Fu	OF	DF	5-Fu	OF	DF
NYL-JN-035*	145.53	372.82	580.66	4.17	5.40	7.03
NYL-JN-036*	1.56	3.02	7.68	0.15	0.56	2.54
NYL-JN-038*		2063.75	2063.75		9.62	10.05
NYL-JN-039*	123.44	142.73	133.52	1.26	2.42	1.85
NYL-JN-040*	4.83	8.86	5.67	6.21	6.97	6.88
NYL-JN-042*	10.93	13.01	13.55	2.83	3.64	3.85
NYL-JN-043*	709.68	723.62	710.37	2.72	3.52	3.23
NYL-JN-049	18.98	30.46	20.97	2.76	4.23	4.03
NYL-JN-051	248.14	1,317.63	239.51	5.85	7.29	6.24
NYL-JN-055	10.56	15.65	17.86	3.28	3.69	3.48
NYL-JN-056	205.38	521.86	372.19	5.78	7.47	6.27
NYL-JN-066		101.42	65.99		5.80	4.59
NYL-JN-067		76.88	164.89		4.57	4.10
NYL-JN-071		17.35	17.35		1.18	1.69
NYL-JN-078		92.72	221.30		5.68	5.74
NYL-JN-079		18.24	27.27		3.62	3.79
NYL-JN-082		10.89	16.07		2.98	3.43
NYL-JN-085		6.55	13.62		1.80	3.28
NYL-JN-087		62.58	43.35		4.72	4.11
NYL-JN-095		6.97	15.79		1.03	2.16
NYL-JN-099		241.95	223.75		7.02	7.42
NYL-JN-110		27.80	65.35		3.30	4.16
NYL-JN-111		88.88	19.53		5.03	4.13
NYL-JN-112		3,366.08	1770.83		9.36	9.39
NYL-JN-113		335.90	336.34		7.15	7.53
NYL-JN-114		1,830.39	1422.81		8.65	9.80
NYL-JN-116		66.40	79.47		5.31	5.63
NYL-JN-117		63.74	61.16		5.54	5.64
NYL-JN-118		80.05	176.40		5.90	6.68
NYL-JN-120		44.50	21.12		5.02	5.67
NYL-JN-125		199.23	246.98		5.38	6.54
NYL-JN-128		557.60	557.6		7.42	7.13
NYL-JN-129		11.24	20.03		2.57	3.83

OF, oxaliplatin + 5-Fu; DF, docetaxel + 5-Fu; ^*^represents gastroscopy specimen patients.

In order to quantify the culture results, the tumor stage of patients, pathological differentiation, and chemotherapy regimens were statistically compared across sources of culture specimens (**Table 3**). In the surgical specimens, the degree of pathological differentiation was a statistically significant driver of culture success. SRC ± G3 had the highest culture success rate and was statistically significant (*P* = 0.028). Other comparisons did not reach statistical significance.

**Table 3:** Cultivation of GC cells using i-CR system across different specimens.

	Surgical specimens		P-value	Gastroscopy specimens		P-value
	SN (%)	NSN (%)		SN (%)	NSN(%)	
Staging						
I	2 (7.69)	5 (10.87)	0.768	1 (14.29)	0 (0.0)	0.438
II	8 (30.78)	9 (19.57)		0 (0.0)	0 (0.0)	
III	16 (61.53)	31 (67.39)		4 (57.14)	8 (57.14)	
IV	0 (0.0)	1 (2.17)		2 (28.57)	6 (42.86)	
Pathological differentiation						
Gx	0 (0.0)	0 (0.0)	0.028	3 (42.86)	3 (21.43)	0.176
G1	0 (0.0)	0 (0.0)		0 (0.0)	0 (0.0)	
G2	5 (19.23)	11 (23.91)		2 (28.57)	1 (7.14)	
G3	12 (46.15)	31 (67.39)		2 (28.57)	10 (71.43)	
SRC ± G3	9 (34.62)	4 (8.70)		0 (0.0)	0 (0.0)	
Chemotherapy regimen						
5-FU	0 (0.0)	0 (0.0)		0 (0.0)	0 (0.0)	
5-FU+ oxaliplatin	9 (34.62)	0 (0.0)		3 (42.86)	0 (0.0)	
5-FU + docetaxel	17 (65.38)	0 (0.0)		4 (57.14)	0 (0.0)	

SN, success number; NSN, no success number; G1, well-differentiated adenocarcinoma; G2, moderately differentiated adenocarcinoma; G3, poorly differentiated adenocarcinoma; SRC, signet ring cell carcinoma. The choice of chemotherapy regimen was based on the MI and DSI results.

### Comparison of i-CR Drug Sensitivity Tests With Clinical Outcomes of GC Patients

WES suggested that i-CR system could be an excellent *in vitro* tumor model for drug sensitivity. We next examined its clinical predictive value for responses to chemotherapeutic agents of GC patients. Four patients underwent neoadjuvant chemotherapy as established by the i-CR system. Since the i-CR culture needed about two weeks to complete, the first cycle of chemotherapy was an empirical medication, but the second through fourth cycles were based on the experimental drug sensitivity results.

**Figure 6:**
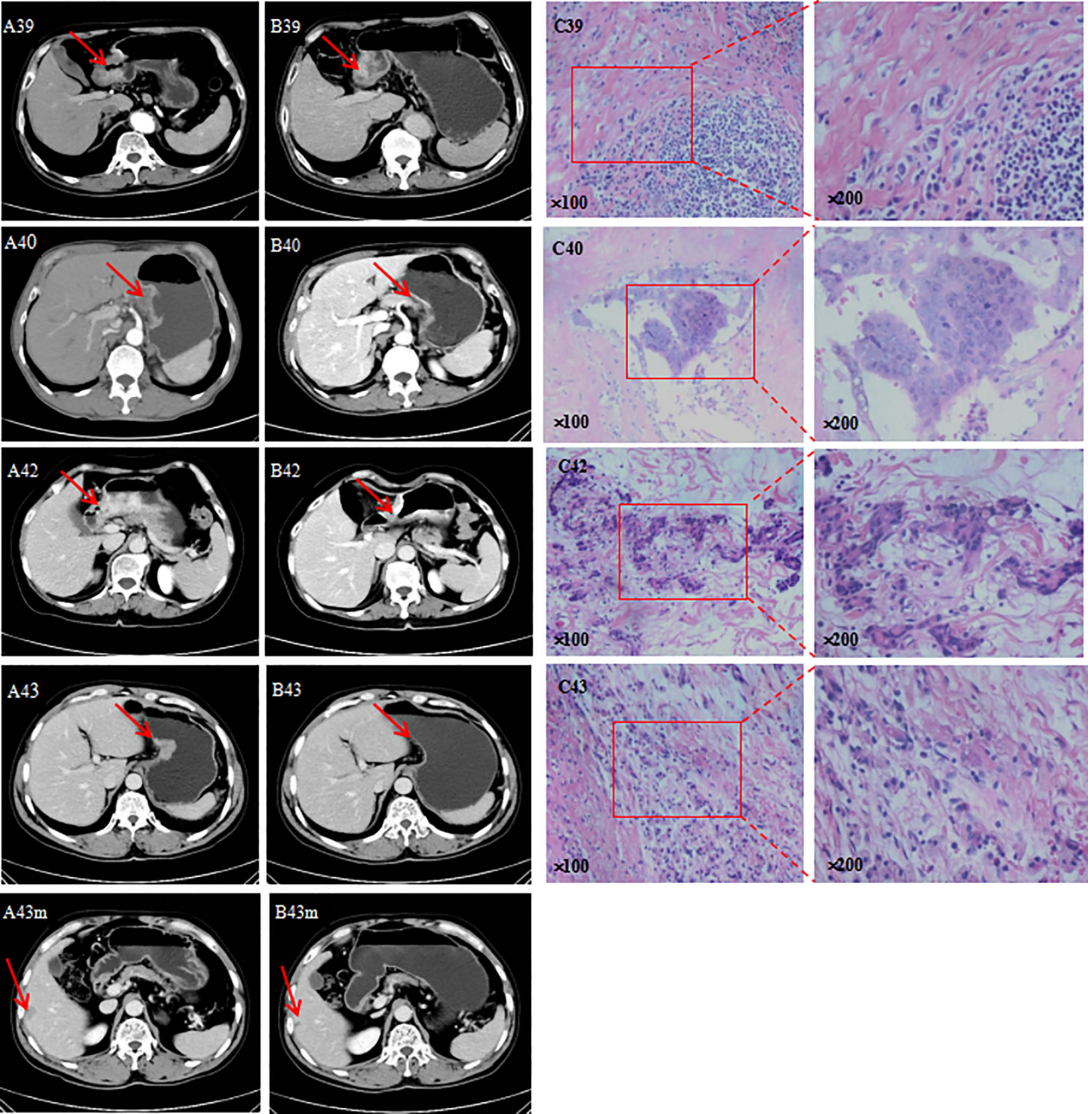
**(A)** CT before neoadjuvant chemotherapy; **(B)** CT after neoadjuvant chemotherapy; m, liver metastasis; **(C)** image pathology after neoadjuvant chemotherapy.

According to analysis of tumor markers, CEA, CA19-9, CA72-4, and AFP showed different degrees of decline, of which CEA was the most sensitive (where 75% of patients had a decline) (**Table 4**). Based on the imaging analysis, three cases were evaluated as PR, and one case was SD. Based on the TRG analyses, two cases were assigned degree 1, one case was assigned degree 2, and one case was assigned degree 0 (**Figure 6**).

**Table 4:** Efficacy of neoadjuvant chemotherapy on patients.

Patients	Tumor Markers								RESIST TRG	
	CEA		CA19-9		CA72-4		AFP			
	BC	AC	BC	AC	BC	AC	BC	AC		
NYL-JN-039	189.2	111.4	17.0	28.68	2.51	2.72	1.94	3.44	SD	1
NYL-JN-040	12.71	4.19	3.28	12.11	3.26	3.83	3.08	3.43	PR	1
NYL-JN-042	1.17	1.63	<0.60	<0.60	5.13	3.96	4.34	3.57	PR	2
NYL-JN-043	3.21	2.70	7.39	10.80	3.72	2.53	4.58	4.36	PR	0

## Discussion

GC is a gastrointestinal malignant tumor that is common in China. Current treatments are comprehensive and incorporate surgery, chemotherapy, and radiotherapy. Due to the high degree of heterogeneity of GC, however, individualization differences are large, there are many chemotherapy options, and effective biomarkers are lacking. Therefore, the effects of chemotherapy are often poor, especially for neoadjuvant chemotherapy patients. Because of these poor effects, chemotherapy resistance, tumor progression, loss of radical surgery as an option, and resource waste are all common (19). Patients often acquire chemotherapy resistance after recurrence and metastasis, and choosing late-line treatments is also difficult. Thus, formulating individualized GC chemotherapy regimens is an urgent issue.

Sensitively and drug resistance of tumor chemotherapeutics are related to many factors, including tumor heterogeneity, immune depletion, tumor cell membrane proton pumps, and the emergence of new phenotypes of tumor cell resistance (20). At present, the most internationally recognized *in vitro* model for tumor growth is the PDX model. This model implants a small piece of tumor tissue taken from a patient into experimental mice to simulate its original growth environment and retain the original tumor characteristics. However, the PDX model has low tumor formation rate, long methodological cycle, high cost, and low clinical patient benefit rate (21, 22). CR of primary tumor cells is a new type of *in vitro* culture system that combines a feeding cell system and ROCK inhibitors. Research on the CR system, especially its application in colorectal cancer, bladder cancer, and prostate cancer cells, suggests that it has great potential for anti-tumor therapeutics (13, 23, 24). A former study found that gene expression profiles of the cell banks in the early stage of patient reprogramming were similar to those of the tumor tissue of the patient, and that different subclones of tumor cells could be amplified indiscriminately in a short time using this system. Our research also showed that P0 and P5 cells showed highly similar SNV and tumor-related gene expression. Genetic analysis based on WES and CNVs suggested CR cells retained the heterogeneity of the original tumor cells. These findings are consistent with related WES and CNV reports that have shown that CR cells maintain tumor heterogeneity (15, 24, 25).

With the optimization of a new generation of culture technology, the emergence of i-CR system has improved the culture efficacy and sensitivity of drug sensitivity applications. Based on the use of this technology platform in colorectal cancer culture and drug sensitivity screening, we learned that culture, selection of culture media, optimization of drug sensitivity formulas, and simulation of steady-state drug concentrations were important factors when applying the system (14). Here, we successfully applied the i-CR system to GC for the first time. Our data suggest that the i-CR system gradually matured in GC *in vitro*. We collected 93 GC specimens. Seventy-two of these were surgical, and we cultured 26 of them successfully. The other 21 cases were gastroscopy specimens, and were cultured seven of them successfully. Among these, 20 cases of the PDX model were established using surgical specimens, and no PDX model was established using gastroscopy specimens. We successfully established a primary tumor cell bank and tested a total of 33 cases, with a success rate of 35.48%. A total of 20 cases of PDX models were successfully established with a success rate of 21.51%. SRC ± G3 had the highest culture success rate. This success may be due to its high degree of malignancy, although the specific mechanism is unknown. The main reasons for the low culture rate of gastroscopy may include bacterial contamination, the overgrowth of benign epithelial cells, and the lack of proliferation caused by the small amount of specimens (23, 26, 27).

Based on the results of the drug susceptibility tests, we performed systematic postoperative adjuvant chemotherapy and neoadjuvant chemotherapy on 33 patients who were successfully tested.

Based on these results, we may infer that adjuvant chemotherapy is a preventive adjuvant chemotherapy. However, there is no clear short term evaluation index, and further evaluation is needed of the indicators of long-term survival rates. For neoadjuvant chemotherapy patients, the results of our treatment evaluation show that the effect is definite. Other traditional evaluation methods (such as tumor markers, imaging and TRG) will need to be used to confirm the consistency of this technology, as well as its clinical utility.

There are also some problems inherent to neoadjuvant chemotherapy. Because our testing cycle is two weeks, the first cycle of neoadjuvant therapy may not align with the results of the drug sensitivity tests. Often the second cycle of treatment is synchronized with the drug sensitivity test, however. This may have impacted our research results. In addition, the combined use of the two chemotherapy drugs has a significantly better inhibitory effect on tumors than either drug dose alone. In most test cases, 5-FU + docetaxel, a clinical standard regimen, has the best inhibitory effects, but there are also individual cases which are more sensitive to 5-FU + oxaliplatin. This reflects the value of individualized precision medicine for GC patients. In theory, the effects of three-drug combination chemotherapy are better than those of two-drug combination chemotherapy, but the general conditions of patients are more demanding. If the interactions between drugs can be further clarified, precision treatment may be further improved. Additionally, there are significant differences in the sensitivity of different patients to drugs within the same tumor type. This “individual difference” causes complexity within tumor drug treatment (28, 29). The i-CR system used in this study is a drug susceptibility detection technology that directly focuses on tumor cell functions. It ignores genetic- and molecular-level changes and directly investigates the response of tumor cells to drugs. The results obtained are compared with the results of clinical medication, which is more precise and accurate.

In summary, this research is based on the concept of individualized and precise GC treatment. Here, for the first time, we combined chemotherapy with an advanced drug sensitivity test platform to provide each GC patient with an effective individualized treatment plan.

There are many strengths to this approach. First, i-CR technology does a good job of representing the growth characteristics of tumors *in vivo*. Compared with conventional cell line cultures, it allows different subtypes of tumor cells to proliferate indiscriminately. This preserves tumor heterogeneity, helping to obtain more accurate drug sensitivity results. Drug sensitivity results based on the i-CR system can also provide accurate support for clinical drug use, and drug susceptibility results are relatively consistent with clinical efficacy. In subsequent research, we will plan to optimize the methods of obtaining specimens, (and particularly to increase the culture rate of gastroscopic specimens and strive to establish a PDX model), and to expand the scope of drug sensitivity (including incorporating three-drug combinations or combinations of targeted therapy drugs). In addition, we will expand in-depth research based on the PDX model, *in vitro* drug screening data, clinical test results and high-throughput omics. This will not only lead to individualized and precise treatments, but will also help identify new biomarkers and drug targets. We ultimately aim to establish a precision medicine research and development platform.

## Data Availability Statement

The datasets presented in this study can be found in online repositories. The names of the repository/repositories and accession number(s) can be found in the article/**Supplementary Material**.

## Ethics Statement

The studies involving human participants were reviewed and approved by Ethics Committee of Shandong Cancer Institute. The patients/participants provided their written informed consent to participate in this study. The animal study was reviewed and approved by Ethics Committee of Shandong Cancer Institute.

## Author Contributions

All authors made a significant contributions to the reported work, including in the conception, study design, execution and/or interpretation; participated in drafting, revising or critically reviewing the article; gave final approval of the version to be published; agreed on the journal to which the article has been submitted; and agreed to be accountable for all aspects of the work. WZ and KL are responsible for clinical data collection. ZS and XQ are responsible for data compilation and statistics. LW, BL, and LL are responsible for collecting clinical specimens. ZC is responsible for basic experiments and drug sensitivity test. JS and JC are responsible for experimental design and technical guidance. All authors contributed to the article and approved the submitted version.

## Funding

This work was supported by Key Research and Development Project of the Shandong Province of China (NO.2019JZZY011008) and Science and Technology Plan Project of Jinan (NO.201907097). The funder was not involved in the study design, collection, analysis, interpretation of data, the writing of this article, or the decision to submit it for publication.

## Conflict of Interest

Author ZC was employed by Beijing Percans Oncology Co. Ltd.

The remaining authors declare that the research was conducted in the absence of any commercial or financial relationships that could be construed as a potential conflict of interest.
