# Supplementary material for: Application Research of Individualized Conditional Reprogramming System to Guide Treatment of Gastric Cancer
Source: Front Oncol. 2021 Jul 16;11:709511. doi: 10.3389/fonc.2021.709511 (PMC8322696; doi:10.3389/fonc.2021.709511)
Supplement: Supplementary file 1 [file DataSheet_1.docx]

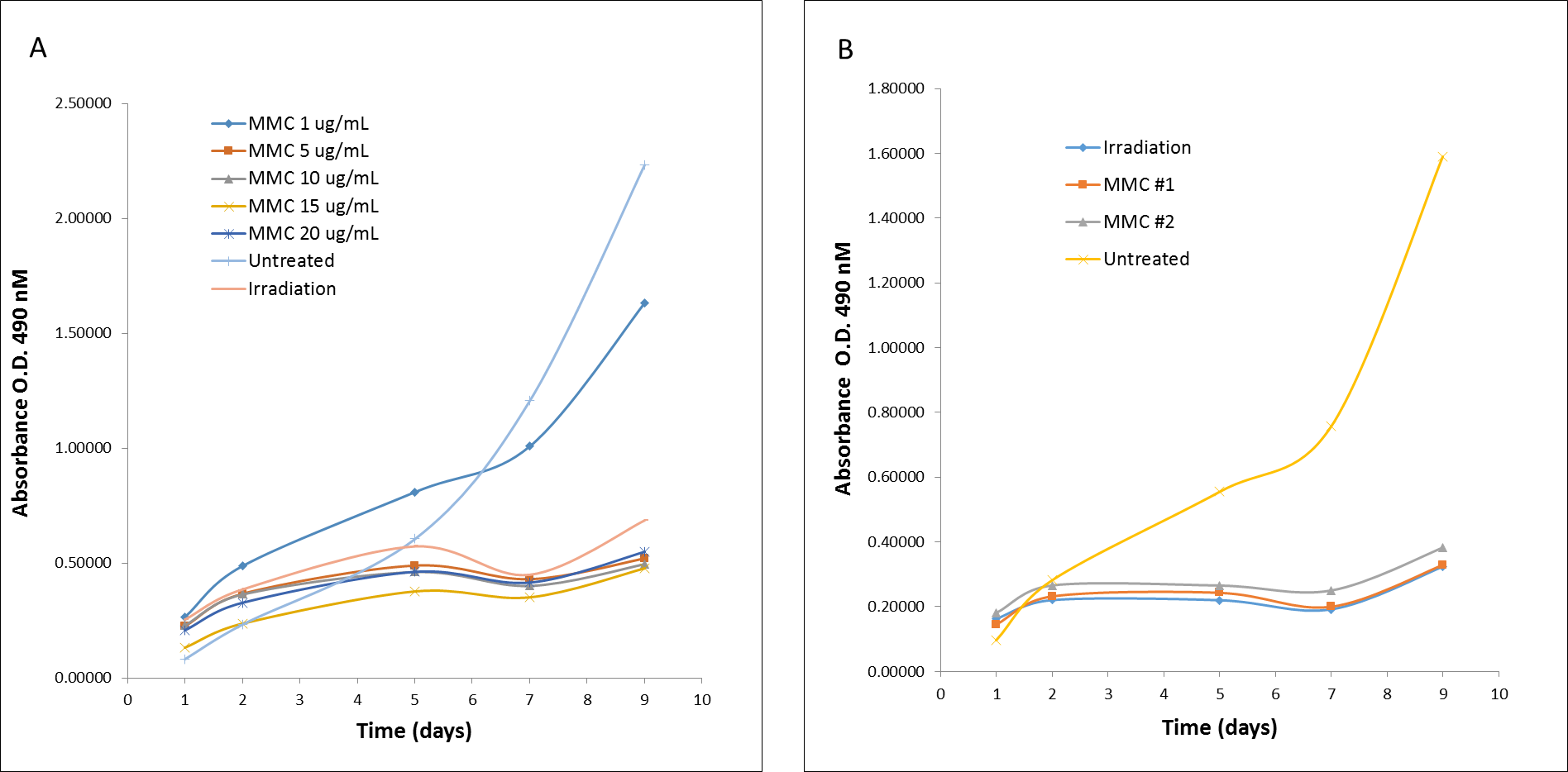


Figure S1. Growth curves of feeder cells. A. NIH3T3 cells were treated with mitomycin C (MMC) at concentrations from 1 μg/mL to 20 μg/mL. The cell proliferation was monitored with MMT method. B. Two batches of feeder cells (MMC #1 and #2), both were treated with 10 mg/mL mitomycin C, were checked for their growth. Irradiated (40 Gy) cells were used as a comparison.


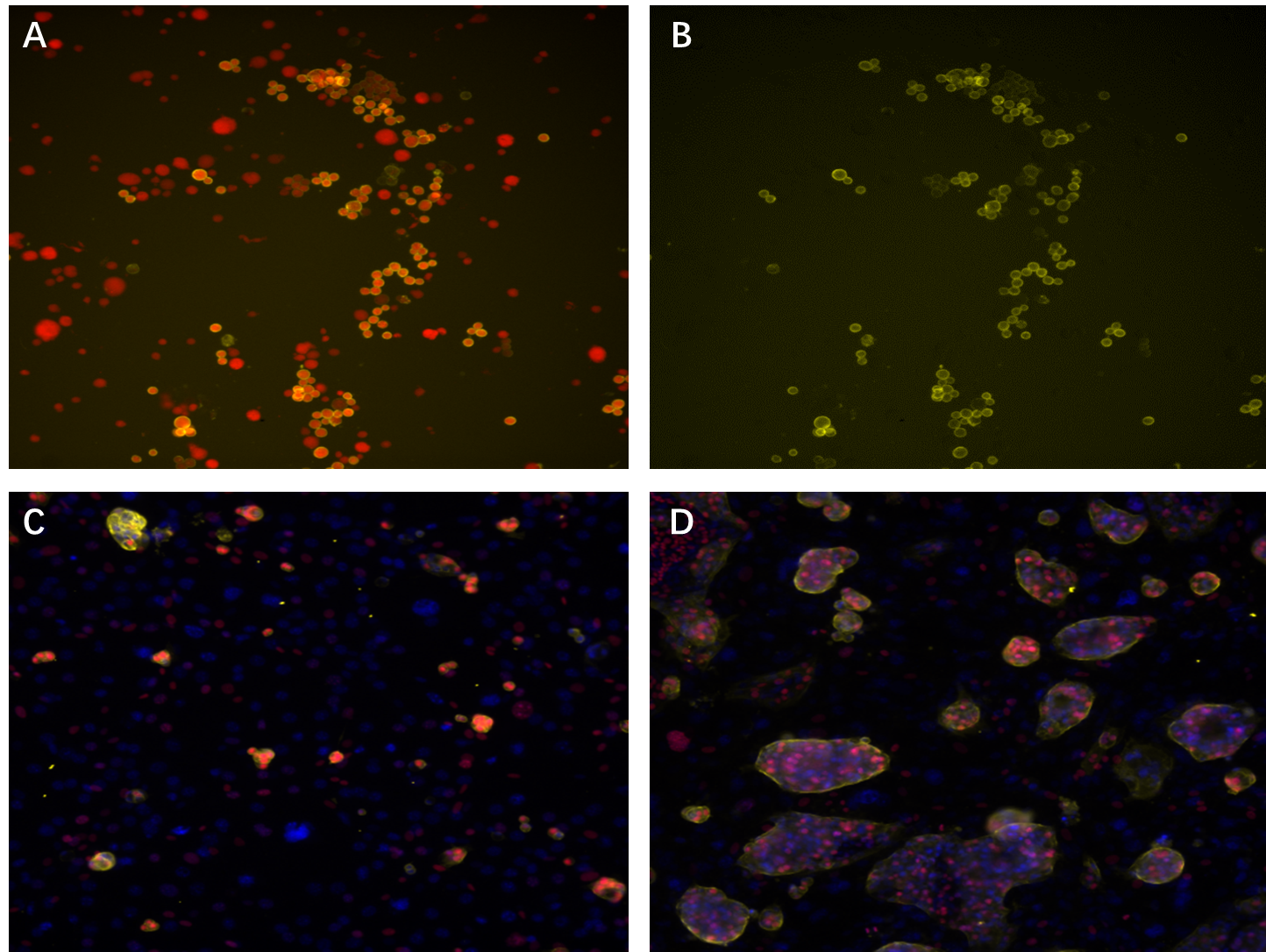


Figure S2. Cell staining during isolation and drug testing. A and B are cells isolated from tumor biopsy sample. The red stain is casein AM and yellow stain is EpCAM. C and D are plated i-CR cells for drug testing. C is at the second day after plating and D is the seventh day. The stains are EdU (red), EpCAM (yellow) and Hoechst (blue).
